# Genotype frequency and use of single nucleotide polymorphisms for detection of informative allele by polymerase chain reaction

**DOI:** 10.12669/pjms.36.7.2998

**Published:** 2020

**Authors:** Ayesha Nayyar, Suhaib Ahmed

**Affiliations:** 1 Dr. Ayesha Nayyar, M.Phil. Department of Pathology, Islamic International Medical College, Riphah International University, Islamabad, Pakistan; 2 Prof. Dr. Suhaib Ahmed, FCPS, PhD, Department of Pathology, Islamic International Medical College, Riphah International University, Islamabad, Pakistan

**Keywords:** Single Nucleotide Polymorphism, PCR, Sibling Pairs, Informative allele

## Abstract

**Objective:**

To determine genotype frequency of biallelic single nucleotide polymorphisms and its use in detection of informative allele in donor/recipient pairs (sibling pairs) having undergone haematopoietic stem cell transplantation with various haematological disorders using a PCR based method.

**Methods:**

This descriptive study was conducted at GRC Lab Rawalpindi from Jan 2018- Oct 2019.A total of twenty donor/ recipient pairs (sibling pairs) were studied for genotype frequency and informativeness of single nucleotide polymorphisms. Genomic DNA was extracted from the peripheral blood and amplification of single nucleotide polymorphisms was done by PCR based method. The amplified DNA was seen by electrophoresis on 6% polyacrylamide gel.

**Results:**

A sharp band of DNA on the polyacrylamide gel indicated a positive reaction. At least two or more informative SNP markers were found in every sibling pair.

**Conclusion:**

Our results demonstrate that PCR amplification of polyacrylamide gel electrophoresis using single nucleotide polymorphism has allowed the successful screening and detection of informative allele in all the donor/recipient pairs. (Sibling pairs). This PCR based assay using SNPs appears to be a quick, simple, reliable and technically feasible method for a use in a Pakistani setting.

## INTRODUCTION

The human genome may contain the most common genetic variation in the form of SNPs.^1^ A single-nucleotide polymorphism may represent as a substitution of a single nucleotide present at a specific location in the human genome. These variations are present in general population with frequency of more than 1%. Any of the two individuals may differ by million SNPs in their genomic pattern.^2^ SNPs sequences has also been utilized in the depiction of population genetics, pharmacogenomics as well as the genes causing cancers and other diseases.^3^

Because of the stability and uniqueness of SNPS, they can be demonstrated by sensitive quantitative assays and have shown to be a useful molecular genetic marker in genetic disease studies^4^ and also helpful in assessing chimerism status after stem cell transplantation in PCR based assays.^5^ This chimerism status involving both donor and recipient patterns are essential in predicting the status of engraftment after stem cell transplantation.^6^ Before stem cell transplantation the donor and recipient genotypes may have to be evaluated by PCR analysis of single nucleotide polymorphism (SNP).

There is no such study available locally which could help in determining the genotype frequency of single nucleotide polymorphisms in donor/recipient (sibling) pairs and their role in a PCR based assay for detection of informative allele in donor/recipient pairs with various hematological disorders. Keeping in view, this study was planned to demonstrate a qualitative PCR based method using SNPs as a genetic marker for detection of informative allele between donor/recipient (sibling) pairs.

## METHODS

It was a descriptive study conducted on twenty sibling pairs selected by non probability convenient sampling from Jan 2018- Oct 2019. The sibling pairs with various haematological disorders having undergone HSCT at the Pakistan Institute of Medical Sciences (PIMS) and Armed Forces Bone Marrow Transplant Center (AFBMTC) were included. The study was approved by the Ethical Review Committee of Islamic international Medical College (Appl. # Riphah /IRC/ 20/202) and informed written consent was taken from the study subjects.

A total of 18 human bi-allelic single nucleotide polymorphisms with high level of heterozygosity were selected from the online database(http://ghr.nlm.nih.gov/handbook/genomicresearch/snp). The sequence of the SNPs was downloaded from the ncbi website (http://www.ncbi.nlm.nih.gov/genbank/) and allele specific primers were designed for each SNP by using the Primer Express (Applied Biosystems, USA) software.

From each subject 2-3 ml venous blood was collected in EDTA. Extraction of genomic DNA was done by the Chelex™ method^7^ (BioRad USA). The reaction conditions for amplification of each SNP by allele specific primers were optimized. A total of twenty sibling pairs were tested at the 18 SNP loci to determine their genotype frequencies and were also tested for the detection of informative allele by using these SNPs.

### PCR Conditions

For SNP-PCR, DNA samples from twenty donor/recipient pairs were tested for these 18 SNPs to identify the informative SNP region.

The lists of primers for amplification of autosomal chromosomal SNPs were as follows:

**Table:** 

Marker Name	Sequence of SNP Primers
S 01a-F	GGTACCGGGTCTCCACATGA
S 01b-F	GTACCGGGTCTCCACCAGG
S 01-R	GGGAAAGTCACTCACCCAAGG
S 02-F	GCTTCTCTGGTTGGAGTCACG
S 02-R	GCTTGCTGGCGGACCCT
S 03-F	CTTTTGCTTTCTGTTTCTTAAGGGC
S 03-R	TCAATCTTTGGGCAGGTTGAA
S 04-F	CTGGTGCCCACAGTTACGCT
S 04a-R	AAGGATGCGTGACTGCTATGG
S 04b-R	AGGATGCGTGACTGCTCCTC
S 05a-F	AAAGTAGACACGGCCAGACTTAGG
S 05b-F	AGTTAAAGTAGACACGGCCTCCC
S 05-R	CATCCCCACATACGGAAAAGA
S 06-F	CAGTCACCCCGTGAAGTCCT
S 06-R	TTTCCCCCATCTGCCTATTG
S 07a-F	TGGTATTGGCTTTAAAATACTGGG
S 07a-R	TGTACCCAAAACTCAGCTGCA
S 07b-F	GGTATTGGCTTTAAAATACTCAACC
S 07b-R	CAGCTGCAACAGTTATCAACGTT
S 08a-F	CTGGATGCCTCACTGATCCA
S 08b-F	GCTGGATGCCTCACTGATGTT
S 08-R	TGGGAAGGATGCATATGATCTG
S 09-F	GGGCACCCGTGTGAGTTTT
S 09a-R	TCAGCTTGTCTGCTTTCTGGAA
S 09b-R	CAGCTTGTCTGCTTTCTGCTG
S 10a-F	GCCACAAGAGACTCAG
S 10b-F	TTAGAGCCACAAGAGACAACCAG
S 10-R	TGGCTTCCTTGAGGTGGAAT
S 11a-F	TAGGATTCAACCCTGGAAGC
S 11b-F	CCCTGGATCGCCGTGAA
S 11b-R	CCAGCATGCACCTGACTAACA
GAPDH-F	GGACTGAGGCTCCCACCTTT
GAPDH-R	GCATGGACTGTGGTCTGCAA

The PCR was carried out in a 25 μl reaction mixture containing 5pM of each primer, 0.5 units of Taq polymerase (Fermentas, Lithuania), 30 mM of each dNTP (Fermentas Life sciences Lithuania), 10 mM Tris HCl (pH 8.3), 50 mM KCl, 1.5 mM MgCl2, 100 mg/ml gelatin (Sigma, UK), and 0.2 μg of genomic DNA. Thermal cycling was done in 9700 (Perkin Elmer, USA) using 28 cycles of: Denaturation 940C for 30 seconds, Annealing 63°C for 30 seconds, and extension 72°C for one minute. The final extension was done for 3 minutes. The amplified products were loaded on 6% non-denaturing polyacrylamide gels measuring 1 mm x 10 cm x 10 cm on Mini-protean electrophoresis apparatus (Bio-Rad, USA). Electrophoresis was carried out at 150V for 40 minutes. The gels were stained by silver nitrate.

## RESULTS

### Informative SNP loci in the Donor/Recipient pairs (sibling pairs)

**Fig.1:**
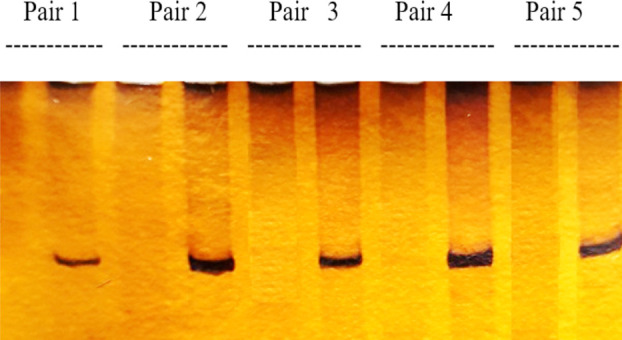
SNP analysis in five sibling pairs at the S07a locus. The marker was informative in all five pairs i.e. one sibling having the “+” allele while the other having the “-” allele.

In each donor/recipient pair an allele was called informative when it was present (+) in one member of the pair and was absent (-) in the other (Fig.1). The frequency of finding an informative allele at any of the SNP locus in the twenty donor/recipient pairs is summarized in Table-I. The number of informative loci in each donor/recipient pair varied from 1 to 10 (Table-I). The informative loci were categorized as “common”, “uncommon”, and “rare” (Table-II). This provided a useful basis for initial screening of informative loci in a new donor/recipient pair. Each pair was initially screened by the “common” loci. The “uncommon” and the “rare” loci were tested when none of the common loci were found to be informative. All of the 20 sibling pairs were discriminated by SNP-PCR. At least two or more informative SNP markers were found in every sibling donor pair (Table-II).

**Table-I:** The results of 18 SNPs in 20 sibling (recipient/donor) pairs. The presence of informative allele is indicated by “+” sign whereas “-“ sign indicates a non-informative marker.

Sibling Pairs	S01	S02	S03	S04a	S04b	S05a	S05b	S06	S07a	S07b	S08a	S08b	S09a	S09b	S10a	S10b	S11a	S11b	Total
Pair 1	-	+	-	-	-	-	-	+	+	-	-	+	-	-	-	-	-	+	5/18 (0.278)
Pair 2	+	-	-	-	-	-	-	-	+	+	-	-	-	-	-	-	-	-	3/18 (0.167)
Pair 3	+	-	+	-	-	+	-	+	+	-	-	-	-	-	-	-	-	-	5/18 (0.278)
Pair 4	-	-	+	+	-	-	-	-	-	-	-	-	-	-	+	-	-	-	3/18 (0.167)
Pair 5	-	-	+	-	-	-	+	-	+	-	-	-	+	-	-	-	-	-	4/18 (0.222)
Pair 6	+	+	+	+	-	+	-	-	-	-	+	+	-	-	-	+	-	-	8/18 (0.444)
Pair 7	-	-	-	+	-	-	-	-	-	-	+	-	-	-	-	-	-	-	2/18 (0.111)
Pair 8	-	-	-	-	+	-	-	+	-	-	-	-	-	-	-	-	-	-	2/18 (0.111)
Pair 9	-	-	-	-	-	-	-	-	+	+	-	-	-	+	-	-	-	-	3/18 (0.167)
Pair 10	-	-	-	-	-	-	-	-	-	+	-	-	-	-	-	-	-	-	1/18 (0.056)
Pair 11	+	-	+	+	-	-	-	-	-	-	-	+	-	-	+	-	+	-	6/18 (0.333)
Pair 12	+	-	-	+	-	+	+	-	+	-	+	+	+	-	+	-	+	-	10/18 (0.556)
Pair 13	-	-	+	-	+	-	-	-	-	-	-	-	-	-	-	-	-	-	2/18 (0.111)
Pair 14	+	+	+	-	-	-	-	-	+	-	-	-	-	-	+	-	+	+	7/18 (0.389)
Pair 15	-	+	-	+	+	+	-	-	-	-	+	-	+	-	+	+	-	-	8/18 (0.444)
Pair 16	-	-	-	-	-	-	-	-	-	-	-	+	-	-	-	-	-	-	1/18 (0.056)
Pair 17	-	-	-	-	+	-	-	-	-	-	-	-	+	+	-	-	-	-	3/18 (0.167)
Pair 18	-	-	+	-	-	-	-	+	+	-	-	-	-	-	+	-	-	-	4/18 (0.222)
Pair 19	+	-	-	-	-	-	-	-	-	+	-	-	-	-	-	-	-	-	2/18 (0.111)
Pair 20	-	-	-	-	-	+	-	-	-	-	-	-	-	-	-	-	-	-	1/18 (0.056)

Total	7/20 0.35	4/20 0.20	8/20 0.40	6/20 0.30	4/20 0.2	5/20 0.25	2/20 0.10	4/20 0.20	8/20 0.40	4/20 0.20	4/20 0.20	5/20 0.25	4/20 0.20	2/20 0.10	6/20 0.30	2/20 0.10	3/20 0.15	2/20 0.10	

**Table-II:** Relative informative-ness of the 18 SNP markers in the 20 sibling (donor/recipient) pairs.

SNP	Informative in sibling pairs	%
*Common*		
S 03	8/20	40.0 (0.40)
S 07a	8/20	40.0 (0.40)
S 01	7/20	35.0 (0.35)
S 04a	6/20	30.0 (0.30)
S 10a	6/20	30.0 (0.30)
*Uncommon*		
S 05a	5/20	25.0 (0.25)
S 08b	5/20	25.0 (0.25)
S 06	4/20	20.0 (0.20)
S 07b	4/20	20.0 (0.20)
S 09a	4/20	20.0 (0.20)
S 08a	4/20	20.0 (0.20)
S 02	4/20	20.0 (0.20)
*Rare*		
S 04b	3/20	15.0 (0.15)
S 11a	3/20	15.0 (0.15)
S 05b	2/20	10.0 (0.10)
S 10b	2/20	10.0 (0.10)
S 11b	2/20	10.0 (0.10)
S 09b	2/20	10.0 (0.10)

##  DISCUSSION

There are number of molecular assays available for genotyping of SNPs e.g. Taqman probe technology,^1^ fluorescence resonance energy transfer probe or molecular beacon methods.^8^ These molecular techniques are generally suitable for large scale assays. For small scale assays, the genotype methods used are gel electrophoresis based, such as denaturing gradient gel electrophoresis (DGGE), conformation sensitive gel electrophoresis and single strand conformation polymorphism (SSCP).^8^

This article describes the informativeness in donor/recipient pairs based on SNP based PCR assay. A panel of 18 human biallelic SNPs clearly discriminated all donor/ recipient pairs by SNP-PCR. In this study the SNP panel for the Pakistani population in a local setting has been optimized. These SNPs were then used in real time PCR for the assessment of donor chimerism in patients with various hematological disorders after hematopoietic stem cell transplantation. Moreover, SNPs with high allele frequency should be identified and selected for other populations and additional SNPs can be easily added for use in different patient groups.

A group of researchers in their study concluded that the informativeness detected by real time PCR assay was 87% of the donor/recipient pairs.^9^ Alizadeh et al used 18 SNPs as genetic markers in 126 DNA samples from 63 recipient/donor pairs. Recipient and donor discrimination was seen in more than 90% of these pairs.^1^

Methods for the analysis of SNPs comprised the allele discrimination between the donor/recipient pairs. Regarding allele discrimination, the most commonly found SNP discriminates 40% of donor/recipient pairs and the least common SNP discriminates 10% of donor/recipient pairs. The 18 SNPs used in this study were able to identify at least one informative marker in all the donor/recipient pairs (100%). A group of researchers have used a panel of 14 bi-allelic SNPs on 55 recipient/donor pairs and concluded that one informative allele was found in all of the pairs tested and two informative alleles were detected in 53 of 55 patient/donor pairs. These researchers suggested that detection rates of informative alleles can be increased many fold by using additional SNPs in donor/recipient pairs.^10^

Jimanez^11^ et al studied on 61 patients of acute leukaemias and found the Informativeness of alleles in 80.3% of donor/recipient pairs. Koldehoff et al investigated 10 different SNP gene regions and were able to discriminate 125 of 135 donor-recipient pairs (92.8%).^12^

Maas^13^ et al has concluded that real time PCR using seven SNP markers were able to quantify the percentage of donor and recipient haematopoietic cells and found out informativeness in 97% of donor/recipient pairs. Similarly, Oliver^5^ et al found that 74% patients showed one informative SNP allele by using 10 SNP genotypes and suggested that 96.5% of recipient/donor pairs will have at least one informative allele by using a panel of 25 SNPs. These findings were in coherence with the panel of 18 SNPs that was tested in this study on the donor/recipient pairs.

## CONCLUSION

Our study showed that SNP-PCR is a useful technique for the detection of an informative allele as a marker of discrimination in sibling pairs with different hematological disorders. Furthermore these informative genetic markers may also be helpful in evaluation of chimerism status in patients having undergone haematopoietic stem cell transplantation for various hematological disorders.

Because our SNP panel is currently optimized for the local Pakistani population, it is likely to require modification for a use in other populations. Importantly, high allele frequency SNPs should be identified for other populations and additional SNPs can be easily added for a use in different patient groups.

### Authors Contribution:

**AN:** literature search, study design and concept, data collection, data analysis, data interpretation, drafting.

**SA:** Study design and concept, data analysis, data interpretation, Critical Review, Final approval.
